# Clinical implications of systolic blood pressure for diabetic retinopathy across HbA1c levels in a Japanese population

**DOI:** 10.1038/s41598-026-35660-w

**Published:** 2026-01-23

**Authors:** Mariko Sasaki, Yoshiko Ofuji, Akiko Hanyuda, Toshihide Kurihara, Yohei Tomita, Kiwako Mori, Nobuhiro Ozawa, Yoko Ozawa, Kazumasa Yamagishi, Kenya Yuki, Norie Sawada, Kazuo Tsubota, Kazuno Negishi, Shoichiro Tsugane, Hiroyasu Iso

**Affiliations:** 1 Department of Ophthalmology, Keio University School of Medicine, 35 Shinanomachi, Shinjuku-ku, Tokyo, 160-8582 Japan; 2 National Hospital Organization Tokyo Medical Center, Meguro-ku, Tokyo, Japan; 3 Department of Ophthalmology, Fujita Medical Innovation Center, Fujita Health University, Ota-ku, Tokyo, Japan; 4 Department of Public Health Medicine, Faculty of Medicine, and Health Services Research and Development Center, University of Tsukuba, Tsukuba, Ibaraki Japan; 5 Ibaraki Western Medical Center, Chikusei, Ibaraki Japan; 6 Department of Public Health, Graduate School of Medicine, Juntendo University, 2-1-1 Hongo, Bunkyo-ku, Tokyo, 113-8421 Japan; 7 Department of Ophthalmology, Nagoya University Graduate School of Medicine, Showa-ku, Nagoya, Japan; 8 Epidemiology and Prevention Group, Research Center for Cancer Prevention and Screening, National Cancer Center, Chuo-ku, Tokyo, Japan; 9 Graduate School of Public Health, International University of Health and Welfare, Tokyo, Japan; 10 Institute for Global Health Policy Research, Bureau of Global Health Cooperation, National Institute for Health Security, Shinjuku-ku, Tokyo, Japan

**Keywords:** Diseases, Endocrinology, Health care, Medical research, Risk factors

## Abstract

This study evaluated the association between systolic blood pressure (SBP) and diabetic retinopathy (DR) across different glycemic levels in a Japanese population. We analyzed data from 1,049 residents aged ≥ 40 years with diabetes who underwent ophthalmic screening in Chikusei City. Participants were stratified into four groups by glycated hemoglobin (HbA1c) level (< 7% or ≥ 7%) and SBP (< 140 or ≥ 140 mmHg). Logistic regression was used to examine associations between SBP and prevalent DR within HbA1c strata. DR was present in 136 participants (13.0%), including 82 (7.8%) with mild-to-moderate nonproliferative DR (NPDR) and 54 (5.1%) with severe NPDR or proliferative DR (PDR). Among participants with lower HbA1c levels, higher SBP was significantly associated with greater odds of DR (odds ratio, 2.21; 95% confidence interval, 1.16–4.23). A similar association was observed among those with mild-to-moderate NPDR, but not among participants with higher HbA1c levels. SBP was not significantly associated with severe NPDR or PDR in any group. These findings suggest that elevated SBP may contribute to the development of early-stage DR even in individuals with well-controlled blood glucose levels, underscoring the importance of blood pressure management in DR prevention strategies.

## Introduction

Diabetic retinopathy (DR), the leading cause of vision loss among working-age adults^1^, is the most common complication of diabetes. A meta-analysis of 59 population-based studies indicated that global prevalence was 22.27% for DR, 6.17% for vision-threatening DR (VTDR), and 4.07% for clinically significant macular edema^2^.

Hypertension, along with hyperglycemia, is a major risk factor for DR^1^. Reportedly, blood pressure (BP) management reduces the risk of DR onset and progression in individuals with type 2 diabetes^3–6^. However, whether the impact of systolic BP (SBP) differs across levels of glycemic control remains unclear. In addition, whether the relationship between SBP and DR risk differs between individuals with well-controlled versus poorly controlled glycated hemoglobin (HbA1c) has not been investigated in most previous studies.

Previous population-based studies have reported inconsistent associations between SBP and severe forms of DR, such as proliferative DR (PDR), diabetic macular edema, and VTDR^1,7–11^. Differences in glycemic status across cohorts, such as the higher HbA1c levels seen in the Los Angeles Latino Eye Study compared with the Singapore Epidemiology of Eye Diseases (SEED) study and the China DiaChronic Study, raise the possibility that the effect of SBP on DR severity may depend on glycemic control^7,10,11^. However, this potential interaction has not been systematically evaluated.

To address these gaps, we examined the cross-sectional associations between SBP and the prevalence and severity of DR, stratifying analyses by blood glucose levels, in a Japanese cohort from the Japan Public Health Center-based Prospective Study for the Next Generation (JPHC-NEXT) Eye Study. This approach allows us to clarify whether BP remains a relevant risk factor for DR under well-controlled glycemia and whether its influence differs by DR stage.

## Results

### Associations between BP and prevalence of DR stratified by HbA1c level

Of the 1,049 participants (610 males and 439 females) with diabetes included in this study, 136 (13.0%) had DR. The participants’ baseline characteristics, stratified by HbA1c level and SBP, are presented in Table 1. Among those with lower HbA1c levels (< 7.0%), those with lower SBP (< 140 mmHg) had a mean SBP of 123.3 mmHg, whereas a mean of 151.6 mmHg was observed for those with higher SBP (≥ 140 mmHg). Among participants with higher HbA1c levels (≥ 7.0%), those with lower SBP had a mean SBP of 124.9 mmHg, whereas a mean of 153.4 mmHg was observed for those with higher SBP. The mean HbA1c levels observed in these four groups were 6.45%, 6.45%, 7.96%, and 8.01%, respectively.

**Table 1:** Baseline characteristics stratified by HbA1c levels and SBP.

	HbA1C < 7, SBP < 140	HbA1C < 7, SBP ≥ 140	HbA1C ≥ 7, SBP < 140	HbA1C ≥ 7, SBP ≥ 140
Variables	No. (%) or Mean (SD)			
N	458	209	254	128
Age, years	68.2 (7.8)	69.4 (7.2)	67.2 (8.8)	67.6 (7.8)
Sex, % male	259 (56.6)	123 (58.9)	150 (59.1)	78 (60.9)
Diabetic retinopathy				
Mild	0	2 (1.0)	5 (2.0)	1 (0.8)
Moderate	14 (3.1)	11 (5.3)	32 (12.6)	17 (13.3)
Severe	8 (1.8)	6 (2.9)	22 (8.7)	14 (10.9)
Proliferative	2 (0.4)	0	1 (0.4)	1 (0.8)
BMI, kg/m^2^	24.0 (3.5)	25.1 (3.4)	24.7 (4.2)	25.3 (3.5)
SBP (mmHg)	123.3 (11.2)	151.6 (9.8)	124.9 (10.4)	153.4 (10.9)
DBP (mmHg)	71.1 (9.2)	82.2 (9.6)	71.4 (9.0)	83.3 (10.7)
Hypertension	10 (2.2)	209 (100)	4 (1.6)	128 (100)
Antihypertensive medication	239 (52.2)	152 (72.7)	135 (53.2)	74 (57.8)
Dyslipidemia	285 (62.2)	140 (67.0)	171 (67.3)	87 (68.0)
Antilipid medication	165 (36.0)	75 (35.9)	99 (39.0)	48 (37.5)
Smoking exposure (pack-years), n (%) (never / <20 / ≥20)	246 (53.7)/ 56 (12.2)/ 156 (34.1)	107 (51.2)/ 22 (10.5)/ 80 (38.3)	120 (47.2)/ 28 (11.0)/ 106 (41.7)	66 (51.6)/ 9 (7.0)/ 53 (41.4)
Diabetes medication, n (%)	313 (68.3)	116 (55.5)	194 (76.4)	98 (76.6)
HbA1c, %,	6.45 (0.35)	6.45 (0.32)	7.96 (1.37)	8.01 (1.23)
Total cholesterol, mmol/L	5.01 (0.89)	5.30 (0.88)	5.11 (0.99)	5.32 (0.96)
HDL- cholesterol, mmol/L	1.48 (0.39)	1.51 (0.36)	1.43 (0.36)	1.47 (0.35)
LDL- cholesterol, mmol/L	2.95 (0.78)	3.18 (0.79)	3.06 (0.86)	3.15 (0.82)
Triglycerides, mmol/L	1.41 (0.76)	1.51 (0.85)	1.49 (0.90)	1.64 (1.06)
Creatinine, mg/dL	0.79 (0.23)	0.80 (0.25)	0.74 (0.24)	0.77 (0.30)

HbA1c, glycated hemoglobin; SBP, systolic blood pressure; BMI, body mass index; DBP, diastolic blood pressure; HDL, high-density lipoprotein; LDL, low-density lipoprotein.

The associations between BP and DR prevalence are presented in Table 2. In the fully adjusted model (Model 2), participants with higher SBP had a significantly higher prevalence of DR than those with lower SBP (odds ratio [OR], 1.73; 95% confidence interval [CI], 1.07–2.77).

**Table 2:** Associations between BP and prevalence of DR stratified by HbA1c levels.

	Number at risk (cases)	Cases, %	OR (95% CI)	
			Model 1	Model 2
SBP < 140	712	84 (11.8)	Reference	Reference
SBP ≥ 140	337	52 (15.4)	1.38 (0.95, 2.00)	**1.73 (1.07**,** 2.77)**
HbA1C < 7, SBP < 140	458	24 (5.2)	Reference	Reference
HbA1C < 7, SBP ≥ 140	209	19 (9.1)	1.82 (0.97, 3.40)	**2.21 (1.16**,** 4.23)**
HbA1C ≥ 7, SBP < 140	254	60 (23.6)	**5.52 (3.34**,** 9.14)**	**5.61 (3.34**,** 9.41)**
HbA1C ≥ 7, SBP ≥ 140	128	33 (25.8)	**6.21 (3.50**,** 10.99)**	**6.26 (3.47**,** 11.29)**

Categories stratified by SBP: Model 1 was adjusted for age and sex; Model 2 was further adjusted for HbA1c, dyslipidemia, serum creatinine, smoking exposure (pack-years), antihypertensive use, lipid-lowering medication use, and diabetes medication status. Categories stratified by SBP and HbA1c levels: Model 1 was adjusted for age and sex; Model 2 was adjusted for the same set of covariates, excluding HbA1c.

BP, blood pressure; DR, diabetic retinopathy; HbA1c, glycated hemoglobin; OR, odds ratio; CI, confidence interval; SBP, systolic blood pressure.

*Significant values are in bold.

Additional subgroup analyses were conducted, stratified by HbA1c level. Among participants with lower HbA1c levels, those with higher SBP had a significantly higher prevalence of DR (OR, 2.21; 95% CI 1.16–4.23) than those with lower SBP. Among participants with higher HbA1c levels, the prevalence of DR was significantly high in both those with lower SBP (OR, 5.61; 95% CI 3.34–9.41) and those with higher SBP (OR, 6.26; 95% CI 3.47–11.29). Notably, among participants with higher HbA1c levels, the prevalence of DR did not significantly differ between those with lower SBP and those with higher SBP (OR, 1.12; 95% CI 0.67–1.86) (not presented in the tables).

### Associations between BP and severity of DR stratified by HbA1c level

Further analysis was performed to examine the relationship between BP and DR severity. The baseline characteristics stratified by DR severity are shown in Table 3. Of the total participants, 82 (7.8%) had mild-to-moderate nonproliferative DR (NPDR), whereas 54 (5.1%) had severe NPDR or PDR. The mean SBP was 132.5 mmHg in participants without DR, 135.3 mmHg in those with mild-to-moderate NPDR, and 138.4 mmHg in those with severe NPDR and PDR. HbA1c level tended to increase across groups, with mean values of 6.9%, 7.3%, and 7.8% in participants without DR, those with mild-to-moderate NPDR, and those with severe NPDR and PDR, respectively.

**Table 3:** Baseline characteristics stratified by severity of DR.

	No DR	Mild and moderate	Severe NPDR and PDR
Variables	No. (%) or Mean (SD)		
N (1049)	913 (87.0)	82 (7.8)	54 (5.1)
Age, years	68.2 (8.0)	68.6 (7.8)	65.5 (8.0)
Sex, % male	525 (57.5)	55 (67.1)	30 (55.6)
BMI, kg/m^2^	24.6 (3.6)	24.9 (3.9)	23.8 (3.7)
SBP (mmHg)	132.5 (16.8)	135.3(18.2)	138.4 (19.2)
DBP (mmHg)	74.8 (10.7)	74.9 (11.7)	76.1 (12.0)
Hypertension	298 (32.6)	31 (37.8)	22 (40.7)
Antihypertensive medication	519 (56.9)	54 (65.9)	27 (50.0)
Dyslipidemia	608 (66.6)	46 (56.1)	29 (53.7)
Antilipid medication	344 (37.7)	29 (35.4)	14 (25.9)
Smoking exposure (pack-years), n (%) (never / <20 / ≥20)	470 (51.5)/101 (11.1)/342 (37.5)	45 (54.9)/ 8 (9.8)/29 (35.4)	24 (44.4)/6 (11.1)/24 (44.4)
Diabetes medication, n (%)	598 (65.5)	78 (95.1)	45 (83.3)
HbA1c, %,	6.9 (1.1)	7.3 (0.9)	7.8 (1.4)
Total cholesterol, mmol/L	5.13 (0.91)	5.03 (1.03)	5.24 (1.12)
HDL- cholesterol, mmol/L	1.47 (0.37)	1.49 (0.35)	1.50 (0.38)
LDL- cholesterol, mmol/L	3.05 (0.80)	3.04 (0.83)	3.09 (0.99)
Triglycerides, mmol/L	1.50 (0.87)	1.19 (0.53)	1.41 (0.99)
Creatinine, mg/dL	0.78 (0.24)	0.81 (0.30)	0.74 (0.23)

DR, diabetic retinopathy; NPDR, nonproliferative DR; PDR, proliferative DR; SD, standard deviation; BMI, body mass index; SBP, systolic blood pressure; DBP, diastolic blood pressure; HbA1c, glycated hemoglobin; HDL, high-density lipoprotein; LDL, low-density lipoprotein.

After full adjustment, SBP was not associated with the prevalence of DR in those with mild-to-moderate NPDR (Table 4). However, when stratified by HbA1c level, among participants with lower HbA1c levels, those with higher SBP had a significantly higher prevalence of DR (OR, 2.51; 95% CI 1.13–5.59) than those with lower SBP. Meanwhile, among participants with higher HbA1c levels, the prevalence of DR was significantly high in both those with lower SBP (OR, 5.78; 95% CI 3.00–11.12) and those with higher SBP (OR, 5.37; 95% CI 2.52–11.41), showing nearly the same ORs.

**Table 4:** Association between BP and severity of DR stratified by HbA1c levels.

	Number at risk (cases)	Cases, %	OR (95% CI)	
			Model 1	Model 2
Mild and moderate NPDR				
SBP < 140	679	51 (7.5)	Reference	Reference
SBP ≥ 140	316	31 (9.8)	1.33 (0.83, 2.13)	1.40 (0.85, 2.29)
HbA1C < 7, SBP < 140	448	14 (3.1)	Reference	Reference
HbA1C < 7, SBP ≥ 140	203	13 (6.4)	2.09 (0.96, 4.53)	**2.51 (1.13**,** 5.59)**
HbA1C ≥ 7, SBP < 140	231	37 (16.0)	**5.91 (3.12**,** 11.19)**	**5.78 (3.00**,** 11.12)**
HbA1C ≥ 7, SBP ≥ 140	113	18 (15.9)	**5.84 (2.80**,** 12.17)**	**5.37 (2.52**,** 11.41)**
Severe NPDR and PDR				
SBP < 140	661	33 (5.0)	Reference	Reference
SBP ≥ 140	306	21 (6.9)	1.47 (0.83, 2.60)	1.72 (0.94, 3.13)
HbA1C < 7, SBP < 140	434	10 (2.3)	Reference	Reference
HbA1C < 7, SBP ≥ 140	196	6 (3.1)	1.44 (0.51, 4.02)	1.91 (0.66, 5.53)
HbA1C ≥ 7, SBP < 140	217	23 (10.6)	**4.97 (2.31**,** 10.68)**	**5.36 (2.44**,** 11.77)**
HbA1C ≥ 7, SBP ≥ 140	110	15 (13.6)	**6.82 (2.97**,** 15.70)**	**7.67 (3.24**,** 18.17)**

Categories stratified by SBP: Model 1 was adjusted for age and sex; Model 2 was further adjusted for HbA1c, dyslipidemia, serum creatinine, smoking exposure (pack-years), antihypertensive use, lipid-lowering medication use, and diabetes medication status. Categories stratified by SBP and HbA1c levels: Model 1 was adjusted for age and sex; Model 2 was adjusted for the same set of covariates, excluding HbA1c.

BP, blood pressure; DR, diabetic retinopathy; HbA1c, glycated hemoglobin; OR, odds ratio; CI, confidence interval; NPDR, nonproliferative DR; SBP, systolic blood pressure; PDR, proliferative DR.

*Significant values are in bold.

For participants with severe NPDR and PDR, SBP was not associated with the prevalence of DR (Table 4). Among participants with lower HbA1c levels, the DR prevalence did not differ between the groups with higher and lower SBP (OR, 1.91; 95% CI 0.66–5.53). Meanwhile, among participants with higher HbA1c levels, the prevalence of DR was significantly high in both those with lower SBP (OR, 5.36; 95% CI 2.44–11.77) and those with higher SBP (OR, 7.67; 95% CI 3.24–18.17). The odds ratio was numerically higher in the higher SBP group than in the lower SBP group; however, this difference was not statistically significant (OR, 1.43; 95% CI 0.70–2.93, not shown in tables).

## Discussion

Population-based studies have consistently shown an association between SBP and DR^1,7–13^. The Los Angeles Latino Eye Study found that higher SBP increased the risk of any DR (per 20 mmHg, OR, 1.26, *P* = 0.002)^7^, and the SEED study reported that SBP was an independent risk factor for any-DR (per 10 mmHg, OR, 1.14; 95% CI 1.09–1.19)^10^. Consistent with these findings, the current study revealed a significant association between higher SBP and the prevalence of DR. Mechanistically, hyperglycemia may impair retinal microvascular autoregulation, increasing endothelial vulnerability to BP-related stress, which can promote capillary injury and retinal nonperfusion, contributing to DR initiation and progression^14,15^.

A unique contribution of this study is the stratified analysis by HbA1c level, which clarifies how the relationship between SBP and DR may differ under varying glycemic control. Among participants with lower HbA1c levels (< 7%), higher SBP was significantly associated with DR, indicating that SBP remains a relevant DR risk factor even when blood glucose levels are well-controlled. However, among participants with higher HbA1c levels (≥ 7%), DR prevalence did not significantly differ across SBP categories, suggesting that the effects of hyperglycemia may overshadow the contribution of BP at higher glycemic levels.

Prior population-based studies have reported inconsistent associations between SBP and severe DR^1,7–11,13,16^ For example, the Los Angeles Latino Eye Study reported that higher SBP was an independent risk factor associated with PDR when compared with NPDR,^7^ suggesting that SBP may contribute more strongly to advanced stages. In contrast, the SEED study and the China DiaChronic Study found comparable effect sizes of SBP for any DR and VTDR,^10,11^ indicating that SBP uniformly influences DR risk across severity levels.

One potential explanation for these discrepancies is the substantial variation in glycemic profiles across cohorts. The Los Angeles Latino Eye Study^7^, for instance, included participants with considerably higher mean HbA1c levels compared with those in the SEED study^10^ and the China DiaChronic Study^11^. Hyperglycemia accelerates cumulative microvascular damage; therefore, elevated BP has been hypothesized to play a more prominent role in the progression to severe or advanced DR with poor glycemic control. Differences in the distribution of blood glucose across cohorts may explain the conflicting findings regarding the impact of SBP on DR severity.

In the current study, the association between SBP and DR varied by glycemic status and disease severity. Among participants with higher HbA1c levels, the association between SBP and mild-to-moderate DR appeared relatively uniform, whereas odds ratios for severe DR were numerically higher among those with elevated SBP; however, these associations were not statistically significant. In contrast, among participants with lower HbA1c levels, the point estimates for the association between SBP and DR were numerically close for mild and severe disease, while the estimate for severe DR was not statistically significant. Given the limited number of severe DR cases, these findings do not reflect definitive conclusions regarding the association between SBP and severe DR, and should be interpreted with caution.

When focusing on mild-to-moderate NPDR, the association between SBP and DR differed by glycemic status. Stratified analyses showed that higher SBP was significantly associated with increased odds of DR among participants with lower HbA1c levels, although no overall association with SBP was observed. This finding suggests that SBP may play a more prominent role in the early stages of DR development when glycemic control is relatively good, highlighting the potential benefit of BP management even in patients without poor glycemic control.

Collectively, these findings indicate that BP management may be particularly relevant for preventing DR onset or early progression, especially among individuals with well-controlled blood glucose levels. In contrast, its role in influencing advanced DR was less evident in the current study, possibly reflecting the dominant contribution of other disease-related factors at later stages. These results underscore the importance of early intervention and support a comprehensive approach to DR risk management that integrates BP and glycemic control across the disease course.

The strengths of this study include the use of standardized grading protocols for DR diagnosis, as assessed by ophthalmologists, including retinal specialists, and detailed questionnaires to collect data on lifestyle and medical history. Nevertheless, some limitations should be acknowledged. First, the cross-sectional design precludes assessment of temporal relationships and raises the possibility of reverse causation. Advanced DR itself is unlikely to directly elevate BP; nevertheless, severe DR may coexist with systemic factors that influence BP regulation. Therefore, the direction of the observed associations cannot be determined. Second, detailed information on the duration of diabetes was not available. Notably, diabetes medication status (newly diagnosed vs. previously diagnosed diabetes) was included as a proxy indicator; however, this measure cannot fully capture the cumulative effects of disease duration. In addition, information on the use of specific antihypertensive drug classes, including renin–angiotensin system blockers, was not available, and antihypertensive use was only adjusted for as a binary variable. Third, the exact timing of BP measurement and the interval since the last use of antihypertensives were not recorded, which may have introduced nondifferential measurement variability. Finally, subgroup analyses stratified by HbA1c level and DR severity included relatively small numbers in some categories, particularly for severe NPDR/PDR, which may have limited statistical precision.

In conclusion, the current study highlights that higher SBP is associated with DR, particularly among individuals with lower HbA1c levels, and may contribute to early disease development. While the effect of SBP on advanced DR remains unclear, our findings underscore the importance of a dual approach incorporating glycemic and BP control for comprehensive DR prevention, particularly in clinical settings where early intervention is feasible.

## Methods

### Study population

The JPHC-NEXT Eye Study is an ancillary study conducted in accordance with the JPHC-NEXT Study protocol^17–19^. In Chikusei City, Ibaraki Prefecture, residents aged ≥ 40 years underwent a systemic and ophthalmological survey. This study included 9,940 individuals who participated in the survey between 2013 and 2017. After excluding 14 participants with missing fundus images or suboptimal image quality (e.g., poor focus, eyelash artifacts, uneven illumination), 1,049 participants with diabetes were included in the analysis.

This study was performed following the Ethical Guidelines for Medical and Health Research Involving Human Subjects in Japan. Approval was granted by the institutional ethics committees of Keio University School of Medicine (Tokyo), University of Tsukuba (Ibaraki), Osaka University (Osaka), and the National Cancer Center (Tokyo). Written informed consent was obtained from every participant before enrollment.

### Data and sample collection

As part of the ophthalmic examinations, nonmydriatic color fundus photographs were captured from both eyes using a 45° nonmydriatic fundus camera (CR-1; Canon Inc., Tokyo, Japan). Each image was centered on the optic disc and macula. In addition, blood samples were obtained to measure serum glucose (fasting or nonfasting), HbA1c (%), total cholesterol (mmol/L), high-density lipoprotein cholesterol (HDL-C) (mmol/L), low-density lipoprotein cholesterol (LDL-C) (mmol/L), triglyceride (TG) (mmol/L), and creatinine (mg/dL) levels. The nonfasting state was defined as < 8 h after the last meal. Diabetes was defined as the use of any antidiabetic medication, a fasting serum glucose level ≥ 7.0 mmol/L, a nonfasting serum glucose level ≥ 11.1 mmol/L, or an HbA1c level ≥ 6.5% (National Glycohemoglobin Standardization Program)^20^. Participants were further classified as previously diagnosed with diabetes if they were using antidiabetic medication; those whose status was identified using laboratory measurements alone were classified as newly diagnosed. To evaluate glycemic control, participants were stratified into HbA1c < 7% and ≥ 7%. The 7% threshold is widely used as the general treatment target for most adults with diabetes, as recommended by the American Diabetes Association Standards of Care^21^. Dyslipidemia was defined as the use of a lipid-lowering medication, an LDL-C level ≥ 3.6 mmol/L, an HDL-C level < 1.0 mmol/L, or a TG level ≥ 1.7 mmol/L^22^. Body mass index was calculated as weight (kg) divided by height squared (m^2^). BP was measured twice using the right upper arm while the participant was seated. The average of the two measurements was used for the analysis. Hypertension was defined as the use of any antihypertensive medication, an SBP ≥ 140 mm Hg, or a diastolic BP ≥ 90 mm Hg^23^. For BP classification, SBP < 140 mmHg and ≥ 140 mmHg were adopted in accordance with the World Health Organization/International Society of Hypertension definition of hypertension^23^.

### Grading of fundus photographs for DR

DR was defined as an Early Treatment DR Study level ≥ 20 in either eye. Its prevalence was determined by two ophthalmologists who were blinded to the participant’s clinical data (T.K., H.T., E.Y., Y.K., K.M., or H.K.). In cases of disagreement, the diagnosis was made by a retinal specialist (Y.T. or N.O.).

### Statistical analyses

Baseline characteristics were summarized and stratified based on HbA1c level and SBP status. In this analysis, HbA1c level and SBP were categorized irrespective of medication use as follows: HbA1c: lower (< 7%) vs. higher (≥ 7%); SBP: lower (< 140 mmHg) vs. higher (≥ 140 mmHg). The associations between BP and DR prevalence were examined using multivariable logistic regression models and expressed as ORs with 95% CIs. For analyses stratified by SBP, the first model was adjusted for age and sex. The second model was further adjusted for HbA1c, dyslipidemia, and serum creatinine levels, as well as smoking exposure (pack-years), antihypertensive use, lipid-lowering medication use, and diabetes medication status (newly diagnosed vs. previously diagnosed diabetes). For categories stratified by SBP and HbA1c levels, the first model was adjusted for age and sex, whereas the second model was adjusted for the same set of covariates, excluding HbA1c to avoid overadjustment. Baseline characteristics stratified by DR severity were calculated for the overall sample. The associations between SBP and DR severity were examined using multivariable logistic regression models. These models were adjusted for the same covariates as those used to examine the associations between BP and DR prevalence. *P*-values < 0.05 were considered statistically significant. All statistical analyses were performed using SAS for Windows, version 9.4 (SAS Institute Inc., Cary, NC, USA).

## Data Availability

The data that support the findings of this study are available on request from the corresponding author, MS or KYa. The data are not publicly available due to their containing information that could compromise the privacy of research participants.
