# Correction: Clinical implications of systolic blood pressure for diabetic retinopathy across HbA1c levels in a Japanese population

**DOI:** 10.1038/s41598-026-47168-4

**Published:** 2026-04-07

**Authors:** Mariko Sasaki, Yoshiko Ofuji, Akiko Hanyuda, Toshihide Kurihara, Yohei Tomita, Kiwako Mori, Nobuhiro Ozawa, Yoko Ozawa, Kazumasa Yamagishi, Kenya Yuki, Norie Sawada, Kazuo Tsubota, Kazuno Negishi, Shoichiro Tsugane, Hiroyasu Iso

**Affiliations:** 1 Department of Ophthalmology, Keio University School of Medicine, 35 Shinanomachi, Shinjuku-ku, Tokyo, 160-8582 Japan; 2 National Hospital Organization Tokyo Medical Center, Meguro-ku, Tokyo, Japan; 3 Department of Ophthalmology, Fujita Medical Innovation Center, Fujita Health University, Ota-ku, Tokyo, Japan; 4 Department of Public Health Medicine, Faculty of Medicine, and Health Services Research and Development Center, University of Tsukuba, Tsukuba, Ibaraki Japan; 5 Ibaraki Western Medical Center, Chikusei, Ibaraki Japan; 6 Department of Public Health, Graduate School of Medicine, Juntendo University, 2-1-1 Hongo, Bunkyo-ku, Tokyo, 113-8421 Japan; 7 Department of Ophthalmology, Nagoya University Graduate School of Medicine, Showa-ku, Nagoya, Japan; 8 Epidemiology and Prevention Group, Research Center for Cancer Prevention and Screening, National Cancer Center, Chuo-ku, Tokyo, Japan; 9 Graduate School of Public Health, International University of Health and Welfare, Tokyo, Japan; 10 Institute for Global Health Policy Research, Bureau of Global Health Cooperation, National Institute for Health Security, Shinjuku-ku, Tokyo, Japan

Correction to: *Scientific Reports* 10.1038/s41598-026-35660-w, published online 23 January 2026

The original version of this Article contained an error. In the Methods section under the Study population subsection, the description of the source population and survey period were incorrect.

As a result,

“In Chikusei City, Ibaraki Prefecture, residents aged 40–74 years underwent a systemic and ophthalmological survey. This study included 7,090 individuals who participated in the survey between 2013 and 2015.”

now reads:

“In Chikusei City, Ibaraki Prefecture, residents aged ≥40 years underwent a systemic and ophthalmological survey. This study included 9,940 individuals who participated in the survey between 2013 and 2017.”

The original Article has been corrected.

